# Protective effects of hydroalcoholic extract from rhizomes of *Cynodon dactylon *(L.) Pers. on compensated right heart failure in rats

**DOI:** 10.1186/1472-6882-9-28

**Published:** 2009-08-05

**Authors:** Alireza Garjani, Arash Afrooziyan, Hossein Nazemiyeh, Moslem Najafi, Ali Kharazmkia, Nasrin Maleki-Dizaji

**Affiliations:** 1 Department of pharmacology and Toxicology; School of Pharmacy, Tabriz University of Medical Sciences, Tabriz, Iran; 2 Department of Pharmacognosy, School of Pharmacy, Tabriz University of Medical Sciences, Tabriz, Iran

## Abstract

**Background:**

The rhizomes of *Cynodon dactylon *are used for the treatment of heart failure in folk medicine. In the present study, we investigated the effects of hydroalcoholic extract of *C. dactylon *rhizomes on cardiac contractility in normal hearts and on cardiac functions in right-heart failure in rats.

**Methods:**

Right-heart failure was induced by intraperitoneal injection of monocrotaline (50 mg/kg). Two weeks later, the animals were treated orally with different doses of the extract for fifteen days. At the end of the experiments cardiac functions and markers of myocardial hypertrophy were measured.

**Results:**

The treated rats showed very less signs of fatigue, peripheral cyanosis and dyspnea. The survival rate was high in the extract treated groups (90%). Administration of *C. dactylon *in monocrotaline-injected rats led to profound improvement in cardiac functions as demonstrated by decreased right ventricular end diastolic pressure (RVEDP) and elevated mean arterial pressure. RV*d*P/*d*t_max_, and RV*d*P/*d*t/P as indices of myocardial contractility were also markedly (p < 0.001; using one way ANOVA) increased by the extract. The extract reduced heart and lung congestion by decreasing tissue wet/dry and wet/body weight ratios (p < 0.01). In the isolated rat hearts, the extract produced a remarkable (P < 0.001) positive inotropic effect concomitant with a parallel decrease in LVEDP.

**Conclusion:**

The results of this study indicated that *C. dactylon *exerted a strong protective effect on right heart failure, in part by positive inotropic action and improving cardiac functions.

## Background

Heart failure is a condition that impairs the ability of the heart to pump a sufficient amount of blood through the body. Treatment strategies have been focused mainly on the use of diuretics and cardiac glycosides. Although, such therapies relieve symptoms of the disease and stabilize patients but rarely improve survival [1]. In addition, pulmonary hypertension represents an important clinical problem that can results in development of right ventricular heart failure [2] and which is associated with a high rate of mortality [3]. It is now believed that medicinal plants can be a valuable source of assistance for prescription medicines and can be taken to aid recovery from serious disease such as heart failure. Digitalis purpurea is an excellent example of herbal use in conventional medicine. Digitalis purpurea contains cardioactive glycosides that effectively treat congestive heart failure [4]. Cynodon species are members of Cynodonteae tribe and Chloridoideae sub-family [5]. *Cynodon dactylon *(L.) pers. (*C. dactylon*; family poaceae), also known as Bermuda grass, Devil's Grass, Couch Grass, Triticum repens, and Indian Doab, is a resilient and perennial grass native to the warm temperate and tropical regions [5,6]. It is populary named "Margh" in Iran. *C. dactylon *is claimed to have antidiabetic, antimicrobial [7], hypolipidemic [8], anti-inflammatory, and anti-emetic [9] properties. The plant is diuretic and traditionally known as a remedy for urinary infections, kidney stones, and congestion. The diuretic effect can be due to the presence of large amount of mannitol in this herb [10]. The root and rhizomes are also used in the treatment of depression, vomiting, cough, epilepsy, and hemorrhage [11]. It has been reported recently that the plant possesses protective effect against stroptozotocin induced hepatic injury in rats [12]. In North West of Iran, *C. dactylon *is named "chayer" and the aqueous extract obtained from its rhizomes is used as a cardiac tonic in heart failure and mostly believed that the extract has very curative effect on heart congestion diseases. The presence of glycosides and flavonoids has been shown in the alcoholic extract of the plant [13] and its curative effect may be attributed to the presence of these phytoconstituents. Recently, we have reported that the total extract of *C. dactylon *rhizomes has a potential protective effect against ischemic/reperfusion injury and improves recovery from the reperfusion insult in isolated rat heart [14]. To our knowledge, the effect of *C. dactylon *on heart failure has not been elucidated to date. Among the most widely studied animal models of right ventricular hypertrophy and failure is the administration of monocrotaline (MCT), which produces a relatively rapid (within a few weeks) pulmonary hypertension resulting in compensatory right ventricular hypertrophy and failure [15,16]. The present study was carried out to investigate the effects of hydroalcoholic extract of *C. dactylon *rhizomes on myocardial responses in right ventricular heart failure as well as on cardiac contractility in isolated normal rat hearts.

## Methods

### Extract preparation

Rhizomes of *C. dactylon *were collected from the fields of Maragheh (Iran) in November and voucher samples were preserved for reference in the Herbarium of Department of Pharmacognosy, School of Pharmacy, Tabriz University of Medical Sciences. Powdered rhizomes (400 g) were extracted by maceration in MeOH/H_2_O (70:30, 3×1 liter) at room temperature for 3 days. The hydroalcoholic extracts were combined and concentrated in vacuo to yield 27 g dried extract. This hydroalcoholic extract was kept in refrigerator for all experiments.

### Phytochemical screening

The crude extract of *C. dactylon *rhizomes was screened for the presence of different classes of compounds using the standard methods by some modifications [17,18]. Thin Layer Chromatography procedures were performed using precoated silicagel plates (Merck; GF_254_, 0.25 mm) to confirm the results of screenings [19]. The following spray reagents were used for detection of respective classes of compounds: Neu's reagent for polyphenoles and flavonoids, Antimony trichloride in chloroform for steroidal saponins and sterols, Kedd reagent for cardiac glycosides, Dragendorrf's reagent for alkaloids, and 5% Ethanolic sodium hydroxide for anthraquinones [19,20]. Since the dried extract contained mainly flavonoid glycosides, it was standardized in its flavonoid glycosides content (4.6%) by the reported method [21].

### Characterization of fractions obtained from the total extract by HPLC

The dried total extract of *C. dactylon *rhizomes was fractionated by solid-phase extraction (SPE) method. The total extract (2 g) was dissolved in minimum possible volume of 20% of MeOH/H_2_O mixture and adsorbed on Sep-Pak (mega tube 10 g; Waters) cartridge and eluted by step gradient of MeOH/H_2_O mixtures (20%; 40%; 60%, 80; and 100%). The high content fractions (20% and 40%), based on weight, were characterized by HPLC finger print. The fractions dissolved in mobile phase and 20 μl of the samples were injected into the reverse phase column (C18). The mobile phase consisted of a gradient of 0–35% (over 35 min), 35–55% (over 15 mhn), and 55-0% (over 10 min) methanol in water (V/V) and was delivered at a flow rate of 1 ml/minute. The detection was performed at three wavelengths of 220, 280, and 350 nm.

### Experimental animals

Male Wistar rats (110–120 g; 4 weeks old) were used in this study. The animals were given food and water ad libitum. They were housed in the Animal House of Tabriz University of Medical Sciences at a controlled ambient temperature of 25 ± 2°C with 50 ± 10% relative humidity and with a 12-h light/12-h dark cycle. This study was performed in accordance with the Guide for the Care and Use of Laboratory Animals of Tabriz University of Medical Sciences, Tabriz-Iran (National Institutes of Health Publication No 85-23, revised 1985).

### Experimental protocol

Animals were randomized into six groups of 10 rats in each. Group 1 (normal control) received an intraperitoneal (i.p) injection of physiological saline (0.5 ml) and untreated for the whole period of the experiment (4 weeks). Group 2 was injected (i.p) a single dose of monocrotaline (50 mg/kg; MCT group) [22] and two weeks later they were given 1 ml vehicle [0.5% carboxy methyl cellulose (CMC)] orally, twice daily. Groups 3 to 6 were injected the same dose of MCT and two weeks later they received digoxin (0.01 mg/kg) or 50, 100, and 200 mg/kg (in 1 ml) of hydroalcoholic extract of *C. dactylon *rhizomes orally, twice daily continued for fifteen days. The extracts were suspended in CMC (0.5%). At the end of the experiments (4 weeks), hemodynamic measurements were taken and then the animals were sacrificed under deep anesthesia, and their hearts and lungs were removed. To assess the direct cardiac effects of the extract, in the other sets of experiments, wistar rat hearts (n = 6) were removed and mounted on a Langendorf apparatus and were exposed to different concentrations of the extract. Left ventricular developed pressure (LVDP), Left ventricular end diastolic pressure (LVEDP), and heart rate (HR) were recorded continuously during the experiment.

### Hemodynamic measurements

Four weeks after MCT injection, the animals were anaesthetized with sodium pentobarbital (60 mg/kg; i.p). When the rats no longer responded to external stimuli, the trachea was cannulated for artificial respiration and systemic arterial blood pressure was recorded from a catheter inserted into the left carotid artery. A standard limb lead I ECG was monitored continuously throughout the experimental period. Mean Arterial Pressure (MAP) was calculated from the systolic and diastolic blood pressures trace. Heart rate (HR) was calculated from the ECG [23]. After recording the above mentioned parameters, the thorax was opened and the right ventricle was punctured with a 23-gauge needle attached to a pressure transducer. After stabilization, right ventricular systolic (RVSP) and end diastolic (RVEDP) pressures were recorded over 5 to 10 min. RV *d*P/*d*t_max _and RV *d*P/*d*t/P as two indices of myocardial contractility were also calculated from RVSP. All parameters were continuously recorded using Powerlab system (AD Instruments, Australia).

### Tissue weights

Following the hemodynamic measurements, animals were sacrificed by an overdose of pentobarbital. The hearts, lungs, livers, and kidneys were removed and weighed. Then, the tissues were cut into small pieces for drying at 55°C until a constant weight was reached. Wet to body weight ratios and wet to dry weight ratios of the tissues were calculated to assess the degree of the congestion.

### Isolated heart perfusion

Rats were anaesthetized with sodium pentobarbital (60 mg/kg intraperitoneally) and given heparin sodium (300 IU). Hearts were rapidly excised and placed in ice-cold buffer and mounted on a constant pressure (100 mmHg) Langendorff-perfusion apparatus. They were perfused with modified Krebs-Henseleit bicarbonate buffer containing (in mM): NaCl 118.5, NaHCO_3 _25.0, KCl 4.8, MgSO_4 _1.2, KH_2_PO_4 _1.2, CaCl_2 _1.7 and D-glucose 12.0. All solutions were gassed with 95% O_2_/5% CO_2 _with pH maintained between 7.35 and 7.45 at 37°C. Temperature was continuously monitored by a thermo-probe inserted into the pulmonary artery and maintained between 36.5 and 37.5°C. A latex, fluidfilled, isovolumic balloon was introduced into the left ventricle through the left atrial appendage and connected to a pressure transducer (P-1000B; Narco Bio Instruments) and HR, LVDP, and LVEDP were recorded with a Narco Bio physiograph (MK-III-S, Narco Bio Systems, USA) [24]. The hearts were allowed to stabilize for 30 minutes before any interventions. Krebs containing different concentrations of the extract was perfused for 1 min at 30 min intervals and maximum responses were recorded. The effects of the extract were expressed as percentage change from pre-perfusion control values.

### Statistics

Data were presented as mean ± SD. Comparisons between groups were made with Student's paired t-test or one way ANOVA as appropriate. If ANOVA analysis indicated significant differences, a Student-Newman-Keuls post test was performed to compare mean values between treatment groups and control. Differences between groups were considered significant at p < 0.05.

## Results

### Phytochemical screening

**Figure 1:**
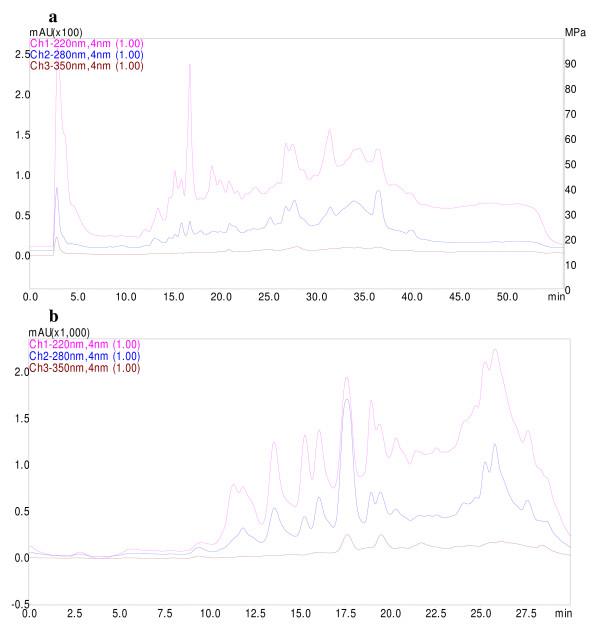
**HPLC fingerprints of fraction 20% (a) and fraction 40% (b) obtained from the total extract of *C. dactylon *rhizomes**. The detection was performed at three wavelengths of 220 nm (upper trace); 280 nm (middle trace) and 350 nm (lower trace).

The finding of preliminary phytochemical screenings showed that the rhizomes of *C. dactylon *contain significant amounts of sugars, flavonoids, sterols and steroidal saponins while, the trace amount of alkaloids were also detectable. The presences of all above mentioned phytoconstituents have been reported previously [13,25,26]. HPLC fingerprints of two fractions (fraction 20% and fraction 40%) obtained from the total extract of *C. dactylon *rhizomes demonstrated different pattern (Figure 1a and 1b).

### General effects of *C. dactylon *extract in rats with right heart failure

Weight gain, which was greatly reduced after MCT administration (Table 1), was significantly high (p < 0.05 and p < 0.01) in the extract treated groups. A week after the administration of MCT, the rats showed a general loss of appetite (food intake reduced from 18 ± 3.3 g per rat to 7.5 ± 4 g per rat 14 days after MCT injection). Water consumption by these animals was also decreased (data not shown). Two weeks after the injection of MCT, the rats showed signs of fatigue and cyanosis of limbs and noses. Although, animals in MCT and MCT plus the extract treated groups showed fatigue and cyanosis, the extract treated rats exhibited an obvious improvement in their appearance and physical activity, even compared to digoxin treated rats. At the end of the experiments, MCT-injected rats (rats with heart failure) showed signs of tachypenea and dyspnea, while these signs were less apparent in the extract treated rats. So that, these symptoms were disappeared in the most of animals treated with 200 mg/kg of the extract. None of the control animals died during experiment period whereas, 2 of 10 rats (20%) from MCT group and groups treated with 50 and 100 mg/kg of the extract died during the experiment period. Mortality was as high as 40% in digoxin treated rats. The group treated with 200 mg/kg of the extract showed less mortality (10%).

**Table 1:** General animal characteristics parameters in the control group and in rats treated with MCT (i.p; Heart failure) or with MCT + *C. Dactylon *rhizomes hydroalcoholic extract (orally).

**Parameter**	**Untreated**		**Treated with Digoxin**		**Treated with *C. dactylon *extract**	
	
	Control	MCT	0.01 mg/kg	50 mg/kg	100 mg/kg	200 mg/kg
**Baseline BW (g)**	151 ± 12	150 ± 10	144 ± 9	147 ± 11	143 ± 9	152 ± 13
**Δ BW (g)**	57 ± 6	26 ± 9^†^	38 ± 11*	32 ± 9	40 ± 6*	43 ± 11**
**Ventricles Wet to Dry weight ratio**	4.35 ± 0.3	5.0 ± 0.13^†^	4.64 ± 0.3*	4.61 ± 0.26*	4.50 ± 0.27**	4.52 ± 0.25**
**Wet ventricle weight to Body weight ratio (g/kg)**	3.31 ± 0.3	4.9 ± 0.7^†^	4.6 ± 0.7	4.9 ± 1.3	4.2 ± 0.8	3.25 ± 0.3**
**Lung Wet to Dry weight ratio**	3.98 ± 0.18	4.98 ± 0.41^†^	4.76 ± 0.21	4.69 ± 0.41	4.70 ± 0.22	4.46 ± 0.25**
**Wet Lung weight to Body weight ratio (g/kg)**	5.01 ± 0.83	8.90 ± 2.9^†^	10.82 ± 2.9	10.03 ± 2.9	8.33 ± 3.4	4.78 ± 0.87**
**Mortality (%)**	0	20	40	20	20	10

Data is expressed as mean ± SD.

Baseline BW, body weights immediately prior to extract administration; Δ BW, increase in body weight over 2-weeks treatment period. All other values obtained 4 weeks after MCT administration. Treatments were started 2 weeks after MCT administration and continued for 2 weeks. † p < 0.01 from respective control value; *p < 0.05; **p < 0.01 from MCT treated group using one way ANOVA.

### Effecst of *C. dactylon *extract on tissue weights

In order to assess the extent of heart hypertrophy developed by the injection of MCT, ventricles wet to dry weight ratio as well as ventricles to body weight ratio were determined (Table 1). Both ratios were significantly higher (p < 0.01) in the rats four weeks after MCT treatment. Compared with MCT-treated rats, treatment with digoxin or the extract produced significant decline in the ventricles wet to dry weight ratio, where this reduction was very notable in groups treated with 100 and 200 mg/kg of *C. dactylon *extract (p < 0.01; Table 1). There was also a decrease in the ventricular to body weight ratio in the extract treated rats, but this reduction reached a significant (p < 0.01) level only by 200 mg/kg of the extract. In order to estimate the extent of proliferative pulmonary responses, wet to dry and wet to body weight ratios of lung was also determined. In comparison with control, wet to dry and wet to body weight ratios of lung were significantly (p < 0.01) high in MCT-injected rats. Compared with MCT group values, only treatment with 200 mg/kg of the extract reduced both ratios of the lungs significantly (P < 0.01; Table 1). There were no significant changes in either the liver or the kidneys wet to dry weight and to body weight ratios with any treatment (data not shown).

### Effects of *C. dactylon *extract on hemodynamic responses

**Figure 2:**
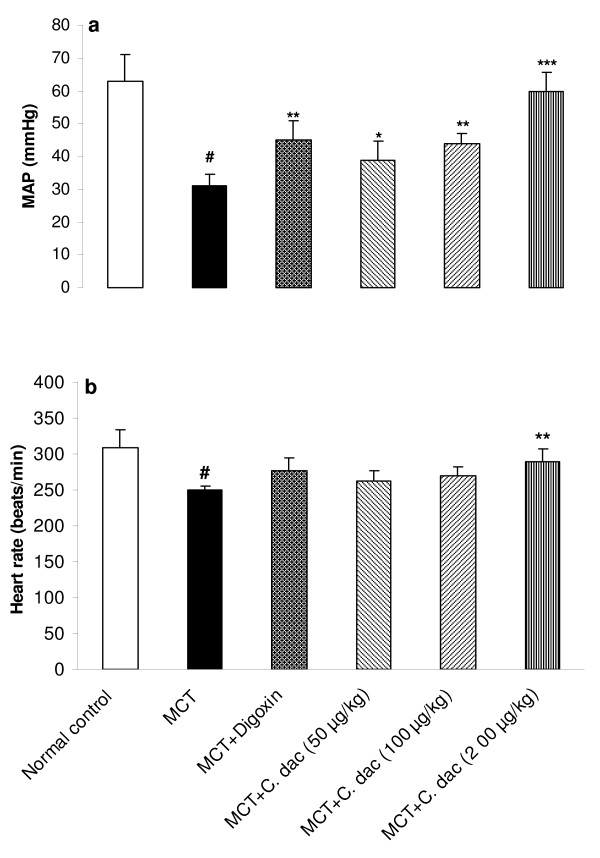
**Mean arterial blood pressure (MAP; a) and heart rate (b) in the normal control group and in rats treated with MCT (heart failure) or with MCT + *C. dactylon *hydroalcoholic extract (*C. dac*)**. Values are mean ± S.D (n = 10). ^#^p < 0.001 from respective control value; *p < 0.05, **p < 0.01, and ***p < 0.001 as compared with MCT treated group using one way ANOVA with Student-Newman-Keuls post hoc test.

**Figure 3:**
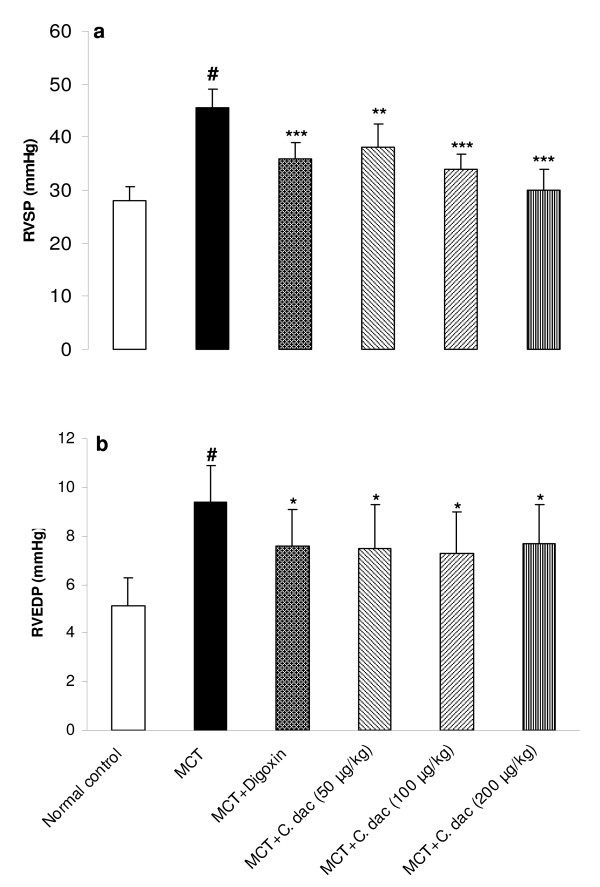
**Right ventricular systolic pressure (RVSP; a) and right ventricular end diastolic pressure (RVEDP; b) in the control group and in rats treated with MCT (heart failure) or with MCT + *C. dactylon *hydroalcoholic extract (*C. dac*)**. Values are mean ± S.D (n = 10). ^#^p < 0.001 from respective control value; *p < 0.05, **p < 0.01, and ***p < 0.001 as compared with MCT treated group using one way ANOVA with Student-Newman-Keuls post hoc test.

**Figure 4:**
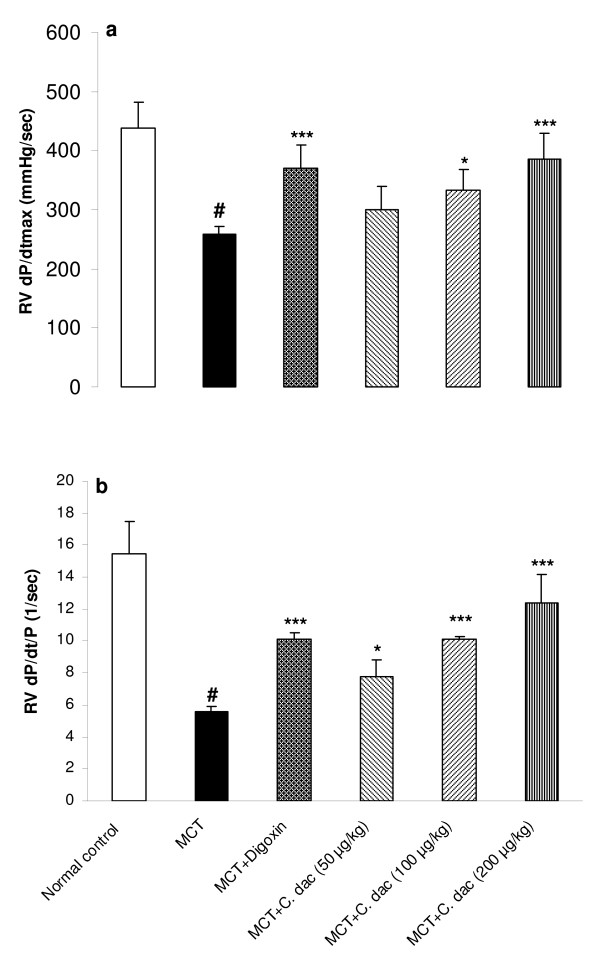
**Right ventricular maximal rate of pressure increase (RV *d*P/*d*t_max_; a) and the rate of pressure change at a fixed right ventricular pressure (RV *d*P/*d*t/P; b) in the control group and in rats treated with MCT (rats with right heart failure) or with MCT + *C. dactylon *hydroalcoholic extract (*C. dac*)**. Values are mean ± S.D (n = 10). ^#^p < 0.001 from respective control value; *p < 0.05, **p < 0.01, and ***p < 0.001 as compared with MCT treated group using one way ANOVA with Student-Newman-Keuls post hoc test.

Mean arterial blood pressure (MAP) was significantly decreased from 63 ± 8 mmHg in control to 31 ± 3.5 mmHg in MCT group (p < 0.001; Figure 2a). Treatment with *C. dactylon *extract prevented the MCT induced reduction of MAP, as 200 mg/kg of the extract reversed the MAP from 31 ± 3.5 (mmHg) close to the control value of 60 ± 5.5 mmHg (p < 0.001; Figure 2a). Heart rate was also significantly (p < 0.001) reduced from 309 ± 25 (beats/min) in control to 250 ± 6 (beats/min) in MCT-injected rats. Treatment with 200 mg/kg of the extract increased the heart rate close to the control value of 290 ± 18 (beats/min; p < 0.01; Figure 2b). To further determine the degree of the right ventricular responses to MCT treatment, intraventricular pressures were measured. RVSP was significantly elevated by 61% (p < 0.001 vs. control) in MCT (heart failure) group. The degree of elevation was significantly attenuated by 50, 100, and 200 mg/kg of the extract to 35%, 21%, and 11%, respectively (p < 0.01 and p < 0.001; Figure 3a). In MCT group, RVEDP was markedly increased by 84% (p < 0.001 vs. control) while, it was lowered with concomitant administration of the extract. The extract with 100 mg/kg reduced this elevation from 9.4 ± 1.5 mmHg in MCT group to 7.3 ± 1.7 mmHg (p < 0.05; Figure 3b). Compared with the normal control, rats with right heart failure (MCT group) demonstrated a lower right ventricular maximal rate of pressure (RV *d*P/*d*t_max_) and lower rate of pressure change at a fixed ventricular pressue (RV *d*P/*d*t/P) (p < 0.001; Figure 4). Similar to the digoxin treated rats, both of these indices of myocardial contractility, were markedly and dose dependently recovered by oral administration of the extract (P < 0.05 and p < 0.001; Figure 4).

### Direct effects of *C. dactylon *extract on isolated rat hearts

**Figure 5:**
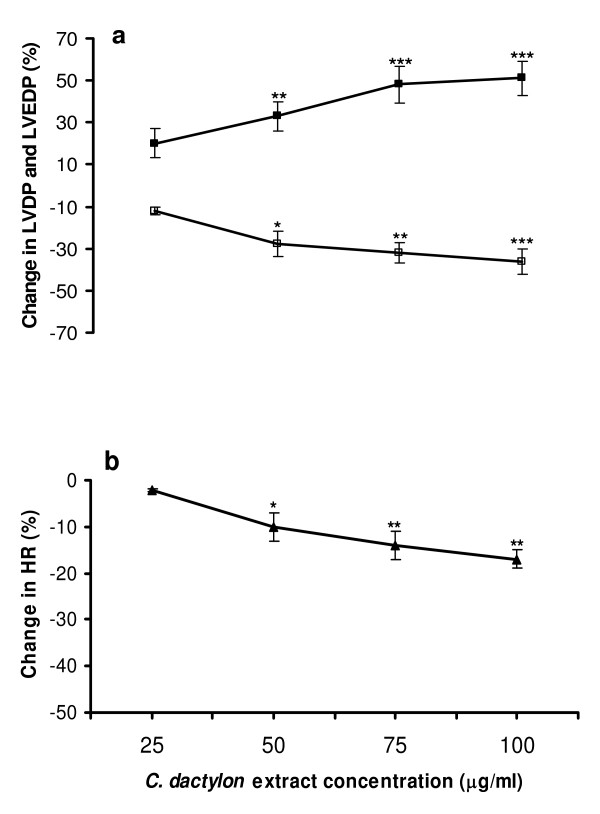
**Direct Effect of the hydroalcoholic extract of *C. dactylon *on left ventricular developed pressure (LVDP; black square; a), left ventricular end diastolic pressure (LVEDP; open square; a), and heart rate (HR; black triangle; b) in isolated rat hearts**. Symbols represent the mean ± SD values (n = 6) of maximum percentage changes in the parameters. *p < 0.05, **p < 0.01, and ***p < 0.001 significantly different from control values using Student's paired t-test.

The extract produced a notable and concentration dependent increase in LVDP. A maximum increase of 51 ± 8% (p < 0.001) was obtained by 100 μg/ml of the extract (Figure 5a). The positive inotropic effect of the extract was concomitant with a parallel decrease in LVEDP (Figure 5a). Likewise, the extract produced a concentration dependent negative chronotropic effect on isolated rat hearts (Figure 5b).

## Discussion

Rhizomes of *C. dactylon *are being used traditionally for the treatment of congestive heart failures in some parts of Iran. However, little is known about its cardioprotective actions in cardiovascular diseases. In the present study, we investigated the therapeutic efficacy of the hydroalcoholic extract of rhizomes of the plant in rats with right heart failure induced by monocrotaline. Monocrotaline produces its effects primarily by injuring pulmonary vessels endothelium, resulting in pulmonary arterial pressure [27]. MCT-induced pulmonary hypertension is associated with development of compensated right ventricular hypertrophy, which progresses to failure within weeks [28]. In this study we started the treatment two weeks after MCT injection when, the heart failure was established. Thus, the results suggest that the favorable effect of *C. dactylon *is mainly due to the inhibition of the compensatory myocardial responses to MCT-induced pulmonary hypertension. The animals on *C. dactylon *treatment fared better than their untreated counterparts and exhibited an obvious improvement in their appearance and physical activity. Symptoms such as cyanosis, tachypenea, and dyspnea were almost disappeared in the most of the animals treated with the high dose of the extract. In addition, the beneficial effects were also clearly observed with respect to a variety of indices, including reduced myocardial hypertrophy, improved cardiac function, positive inotropic action, increase in myocardial contractility force, and parallel decrease in right ventricular end diastolic pressure. Furthermore, direct effect of hydroalcoholic extract of *C. dactylon *rhizomes on isolated rat hearts demonstrated that the extract produce a dose dependent and very significant increase of the contraction force by increasing the left ventricular developed pressure (LVDP) and parallel decrease of end diastolic pressure (LVEDP) and heart rate. In the treatment of heart failure it is very beneficial to improve the contractility of the heart and at the same time decrease the end diastolic pressure. Our preliminary phytochemical finding in this study revealed the presence of sugars, flavonoids, sterols and steroidal saponins in the hydroalcoholic extract obtained from *C. dactylon *rhizomes. Reactive oxygen species have been implicated in the progression of ventricular hypertrophy to congestive heart failure [29]. Plant flavonoids are antioxidant and scavenge oxygen free radicals, therefore suggesting their role in preventing of monocrotaline induced damages in this study. The other main components of the extract are steroidal saponins. Saponins are mainly plant-derived glycosides, occurring as triterpenoid or steroid saponins. Steroid saponins have been found to have some interesting biological and pharmacologic activities including cardiac tonic, diuretic, antibacterial, anti-inflammatory and hypocholesteremic influences [30,31]. Furthermore, the sugar, probably mannitol, and saponins present in the extract have diuretic properties and can be useful in the treatment of congestion disorders such as heart failure.

## Conclusion

The results of this study showed the beneficial effect of *C. dactylon *in rats with compensated right heart failure. We speculate that the extract produce its protective effects at least in part by direct improvement of cardiac performance. However, the precise mechanism for the salutary effect of *C. dactylon *on compensatory myocardial response needs to be determined especially in view of the right ventricular heart failure nature and finding the active ingredient(s).

## Competing interests

The authors declare that they have no competing interests.

## Authors' contributions

AG designed and coordinated the study and also drafted the manuscript. AA carried out the hemodynamic measurements and the heart failure experiments. HN collaborated in the phytochemical procedures. MN helped in the animal experiments and acquisition of data. AK carried out the isolated heart perfusion experiments. NMD has been involved in revising the manuscript and performed the statistical analysis. All authors have read and approved the final manuscript.

## Pre-publication history

The pre-publication history for this paper can be accessed here:


